# Prevalence and Risk Factors of PrEP Use Stigma Among Adolescent Girls and Young Women in Johannesburg, South Africa and Mwanza, Tanzania Participating in the EMPOWER Trial

**DOI:** 10.1007/s10461-022-03721-6

**Published:** 2022-07-01

**Authors:** R. J. Munthali, A. L. Stangl, D. Baron, I. Barré, S. Harvey, L. Ramskin, M. Colombini, N. Naicker, S. Kapiga, S. Delany-Moretlwe, the EMPOWER study team

**Affiliations:** 1 Wits RHI, University of the Witwatersrand, Johannesburg, South Africa; 2 International Center for Research on Women, Washington, DC USA; 3 Hera Solutions, Baltimore, MD USA; 4 University of North Carolina, Chapel Hill, NC USA; 5 London School of Hygiene and Tropical Medicine, London, UK; 6 Mwanza Intervention Trials Unit, Mwanza, Tanzania

**Keywords:** Pre-exposure prophylaxis (PrEP), HIV, Stigma, Sub-Saharan Africa, Adolescent girls and young women (AGYW)

## Abstract

Adolescent girls and young women (AGYW) in sub-Saharan Africa may benefit from pre-exposure prophylaxis (PrEP), yet stigma may limit PrEP acceptance and continuation. We examined factors associated with PrEP use stigma among 307 participants of the EMPOWER trial (2016–2018), an unblinded randomized controlled trial among HIV-negative, AGYW, aged 16–24, in South Africa and Tanzania. The 6-item, brief-PrEP use stigma scale (B-PSS) had high internal reliability. At the end of the trial, 34.2% of study participants reported any PrEP use stigma. Three latent classes were observed, reflecting low (46.9%), medium (31.9%), and high (21.2%) reported PrEP use stigma. Disclosure of PrEP use to sexual partner and belief that PrEP prevents HIV were associated with less reported PrEP use stigma. Conversely, participants who reported fear and shame about people living with HIV were more likely to report PrEP use stigma. Our validated tool and findings will enable practitioners to identify AGYW at high risk of PrEP use stigma who may benefit from additional support.

*Pan African clinical trials registry* PACTR202006754762723, 5 April 2020, retrospectively registered.

## Introduction

Adolescent girls and young women (AGYW) aged 15–24 bear a disproportionate burden of HIV in sub-Saharan Africa (SSA) [1] and may benefit from access to female-controlled biomedical prevention methods [2, 3]. Pre-exposure prophylaxis (PrEP), including oral PrEP with tenofovir (TDF) co-formulated with emtricitabine (TDF/FTC), is one such option that is increasingly being licensed for use in countries worldwide, including across SSA [4]. Yet, stigma associated with PrEP use may pose a barrier to uptake and continuation on PrEP [5, 6], particularly among AGYW [7].

Studies have shown that PrEP is more than 90% effective in preventing HIV when properly taken, although PrEP uptake is variable across studies and continuation remains a persistent challenge [3, 8]. The fear of social harms such as violence [9], rejection or stigmatization [10], may have a profoundly negative influence on AGYW’s ability to incorporate HIV prevention into sexual relationships [11]. Stigma has been defined by sociologist Erving Goffman as a discrediting attribute that leads “a whole and usual person” to be considered a “tainted or discounted one” [12]. PrEP-related stigma is a social harm involving the association of negative meaning with PrEP and corresponding devaluation of PrEP users [13]. As with other health-related stigmas, PrEP-related stigma may manifest as anticipated stigma (e.g. expectations of bias being perpetrated by others if their PrEP use becomes known) or experienced stigma (e.g. the experience of stigmatizing or discriminatory behaviors, such as gossip, violence, relationship dissolution, etc. for using PrEP) among PrEP users [14].

Globally, research has shown that PrEP use may be stigmatized for several reasons, including associations with sex work, same-sex sexual behavior, and drug use, based on context-specific cultural beliefs and HIV epidemiology [15–17]. In Africa, barriers to PrEP uptake and adherence also include the perception that PrEP is for ‘promiscuous’ individuals [7, 18]. Recent qualitative research from SSA suggests that PrEP-related stigma takes two main forms among AGYW: HIV stigma, related to concerns that others will assume AGYW using PrEP are living with HIV, and sexual activity stigma, related to taboos around sexual activity among unmarried women [7].

Several studies have identified HIV stigma, which we term ‘PrEP-HIV stigma’, as a barrier to PrEP use [19, 20]. Antiretroviral therapy (ART)-containing prevention products in particular can lead to discrimination against users [21]. In Kenya and Thailand, participants in oral PrEP trials reported experiencing stigma related to the assumption that oral PrEP was HIV treatment. This led to concealment of product use, and subsequent lower adherence [22, 23]. Similarly in the VOICE trial, PrEP use was linked with HIV illness by participants, their male partners and community members, which led to study product concealment [24].

In some instances, unintentional disclosure of PrEP use in the VOICE trial led to relationship conflicts and early trial termination, due to concerns that PrEP use signified promiscuity [24]. In the EMPOWER trial, some participants were discouraged from using PrEP by their parents, as they worried that PrEP use would lead to disclosure of premarital sex and subsequent stigmatization by community members [25]. We term PrEP stigma related to promiscuity and pre-marital sex, ‘PrEP-sexual stigma’. Participants may have first-hand experience of these PrEP-related stigmas or may anticipate experiencing such stigmas, including fear about others’ negative reactions to and/or perceptions about PrEP [20, 24, 26].

While research suggests that PrEP use may elicit its own specific stigma, few PrEP trials have systematically assessed PrEP use stigma and little is known about the dimensions and correlates of PrEP use stigma among AGYW in SSA [5]. There are currently four validated scales to assess various dimensions of PrEP-related stigma that have been tested among men who have sex with men [27, 28], transgender women [27] and cis-gender heterosexual women in the US [5], but no scales have been tested among AGYW in SSA. Using data from the EMPOWER trial, we sought to explore the dimensions of PrEP use stigma among AGYW in SSA, construct and validate a scale to assess PrEP use stigma, and examine the factors associated with PrEP use stigma among our study population.

## Methods

### Study Design and Population

The EMPOWER trial was an unblinded randomized controlled trial in a population of HIV-negative, PrEP-naïve AGYW aged 16–24. It was conducted in Johannesburg, South Africa and Mwanza, Tanzania between September 2016 and December 2018. At the time of the trial, PrEP was not widely available, and participants were not familiar with PrEP, nor did they have experience using it. Participants were included in the study if they reported current sexual activity, defined as having had vaginal intercourse at least once in the previous 30 days, had regular access to a mobile phone, and were not pregnant at the time of recruitment. Participants (N = 431, South Africa: 379, Tanzania: 52) were then randomized into the standard of care (SOC) (N = 218) or SOC plus EMPOWER clubs (N = 213) arms of the study.

### Intervention and Follow-Up

The SOC package included adherence counselling, text message reminders for visits and adherence, screening, and appropriate referral for gender-based violence, and community dialogues. The EMPOWER clubs included four empowerment sessions delivered from a standardized curriculum [29]. Participants were followed up for up to 6 months in Mwanza, Tanzania and up to 15 months in Johannesburg, South Africa. The main findings are reported in detail elsewhere [30].

Questionnaires were administered by computer assisted self-interview in South Africa and by interviewer administered interview in Tanzania. For the current analysis, only participants with data on PrEP stigma at endline with complete data on the baseline variables were included in the multivariable analyses (N = 307 participants; South Africa: 276 and Tanzania: 31).

### Outcome Measures

**Fig. 1:**
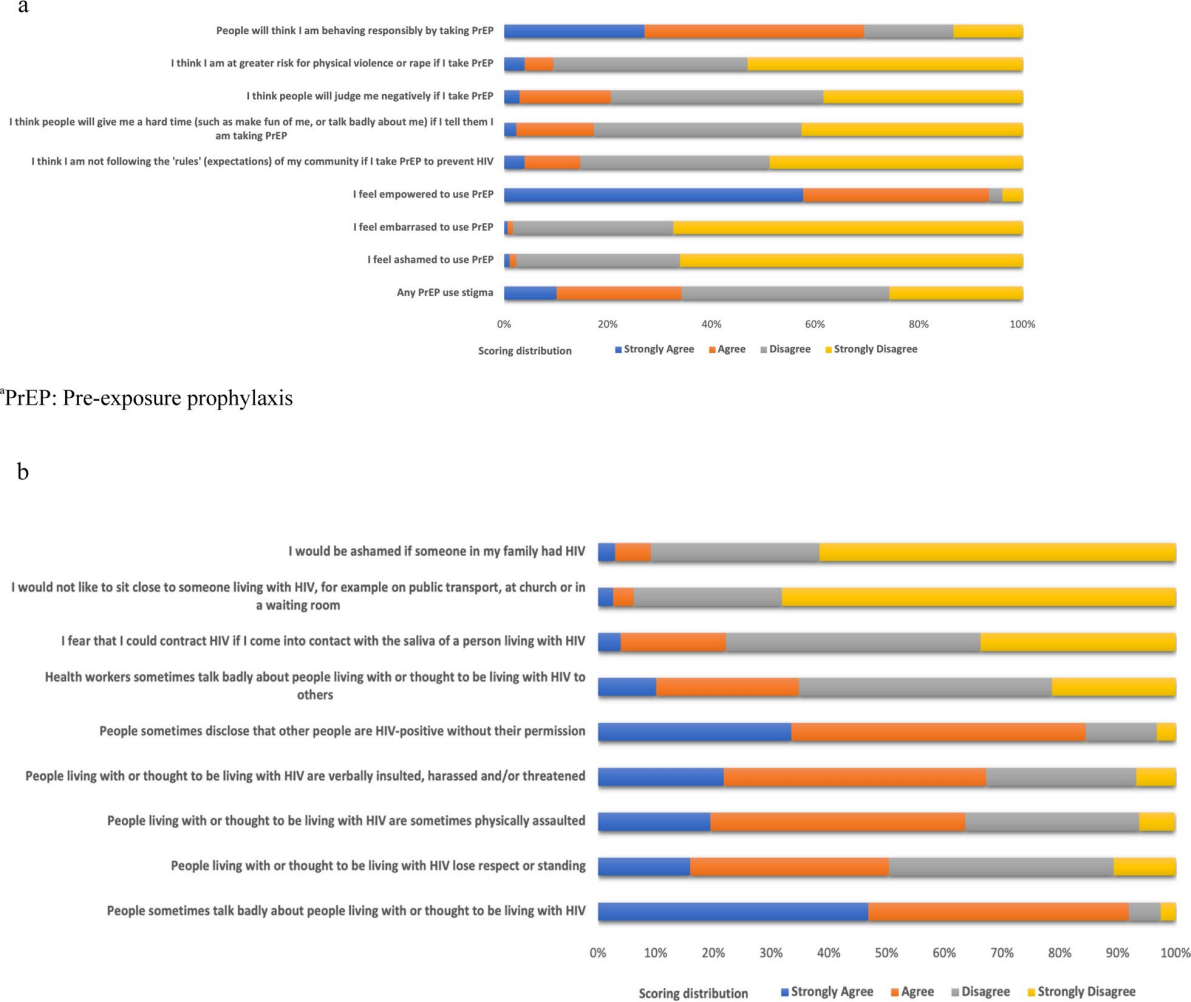
**a** Scoring distribution of the 8 items of PrEP^a^ use stigma assessed. **b** Scoring distribution for the 9 items of HIV stigma assessed

The final 6-item, brief-PrEP use stigma scale (B-PSS) used to assess PrEP use stigma was informed by validated measures used to capture HIV stigma [31], current literature on PrEP stigma [5, 15, 16, 28, 32] and expert consultation with researchers who were also measuring PrEP stigma in other trials. Information on PrEP use stigma was collected from all participants in the EMPOWER trial using eight items that were intended to capture two different aspects of PrEP use stigma. *Personal feelings about PrEP use* were assessed using 4 items (e.g. I feel ashamed of using PrEP; I feel embarrassed about using PrEP, etc.). While *anticipated stigma and discrimination* were assessed using 4 items (e.g. I think people will give me a hard time if I tell them I am taking PrEP; I think I am at greater risk for physical violence or rape if I take PrEP, etc.). PrEP use stigma items were asked at endline only, as the baseline questionnaire was completed before PrEP initiation. All items used a 4‐point agreement rating scale from 0 = “strongly disagree” to 3 = “strongly agree”, with higher scores reflecting more stigma. A dichotomous variable reflecting ‘any PrEP use stigma’ was constructed using responses to 6 items from the final scale (Fig. 1a).

For the multinomial logistic regression analyses of the latent classes of PrEP use stigma identified, we compared membership between the high or medium PrEP use stigma classes with membership in the low PrEP use stigma class, respectively.

### Independent Variables

Independent variables included in the analyses were selected based on factors shown to be associated with PrEP-related stigma in previous research. The baseline values for all potential confounders were used in the unadjusted and adjusted multivariable analyses. Study and sociodemographic characteristics assessed included study site (South Africa, Tanzania), age (16–20, 21–24), marital status (yes/no), number of children (0, 1+), living status (alone/child, parent/relative, partner), and currently in school (yes/no).

Several health risk factors were assessed. Depressive symptoms were captured with the CESD-10, which was recently validated for use in South Africa [33]. Scores greater than or equal to 10 indicate depressive symptoms. HIV risk was measured by the VOICE score [34], with scores greater or equal to 5 suggesting high HIV risk. Participants were also asked about their perceived risk of HIV (not worried, some worry, a lot of worry) and whether they had ever experienced gender-based violence, including physical, sexual or emotional violence [35]. Hazardous and harmful drinking in the last 12 months was assessed using the 10-item AUDIT screening tool [36], which has been validated in numerous settings around the world with numerous populations [37], including adolescents [38]. Scores of 8 or greater reflect hazardous or harmful alcohol consumption. Participants were also asked about their belief that PrEP prevents HIV (yes/no) and whether they had told their partner about their plans to use PrEP (yes/no).

Sexual behavior risk factors assessed included number of sexual partners [39], partner age difference (< 5 years, ≥ 5 years) [40], and any sexually transmitted infection (yes/no). For each sexual partner reported, participants were asked a series of questions adapted from the STRIVE technical brief [41], to assess transactional sex in the last 4 weeks. Agreement with any of the following questions constituted engagement in transactional sex: Did this partner ever give you any gifts, help you to pay for things, or help you in other ways? Did you become sexually involved with this partner because he provided you with, or you expected that he would provide you with gifts, help you to pay for things, or help you in other ways? Would you leave this partner if he no longer provided gifts, helped you to pay for things, or helped you in other ways?

HIV stigma was assessed using nine items from a scale previously validated in South Africa and Zambia [31]. All items used a 4‐point agreement rating scale from 0 = “strongly disagree” to 3 = “strongly agree”, with higher scores reflecting more stigma. We constructed two dichotomous variables that captured agreement with any of the following negative attitudes: ‘I would be ashamed if someone in my family had HIV’, ‘I would not like to sit close to someone living with HIV’, and ‘I fear I could contract HIV if I come into contact with the saliva of a person living with HIV’ and agreement with the following perceived stigma items: ‘health workers sometimes talk badly about people living with HIV’, ‘people sometimes disclose that others are living with HIV without their permission’, ‘people living with HIV are verbally insulted, harassed and/or threatened’, ‘people living with HIV are sometimes physically assaulted’, ‘people living with HIV lose respect and standing’ and ‘people sometimes talk badly about people living with HIV’ (Fig. 1b).

### Statistical Analysis

#### Exploratory Factor Analysis

Exploratory factor analysis (EFA) was conducted to assess the underlying domains of PrEP use stigma. All eight items, Fig. 1a, were included in the initial EFA and the two items that were framed positively were reverse coded. Scree plots and eigenvalues were examined to determine the number of factors to retain based on the number that explained the most variability in the data. Iterated principal factor estimation using oblique (promax) rotation, to allow for correlation between items, was used to examine the loading strength of items on each factor. The internal consistency of each factor was assessed by calculating Cronbach’s alpha and examining item uniqueness. A Cronbach’s alpha value of 0.60–0.69 was considered “acceptable”, 0.70–0.79 “good”, and over 0.80 “very good”, as suggested by Nunnally [42].

#### Regression Analysis Predicting PrEP Use Stigma

Chi-squared tests were performed for categorical variables and Wilcoxon rank sum or Student’s *t*-test for continuous variables. Both unadjusted and adjusted multivariable logistic regression were used to test associations between risk factors and the outcome variable, ‘any PrEP use stigma’. Multivariable models were adjusted for the following potential confounders: study site, age range, marital status, living status, currently in school and number of children.

#### Latent Class Analysis

To identify distinct homogeneous latent classes with respect to PrEP use stigma, we used latent class analysis (LCA). Latent classes have been used to capture heterogeneity in patterns of perceived barriers to PrEP use [43] and in other HIV risk and prevention research [5, 6, 16]. To understand the full picture of PrEP use stigma, we used all eight items (Fig. 1a) to capture the homogeneous groups or classes based on how participants responded to all items [44, 45]. We used the classes identified to characterize and group individuals with similar patterns [46]. Participants were assigned to the class for which they had the highest posterior probability of membership. The model fitting utilized maximum likelihood estimation.

The optimal number of latent classes were assessed using: (a) fit statistics (i.e. prioritizing models with lower values for Akaike information criterion (AIC) and Bayesian Information Criterion (BIC) [47], (b) entropy, (c) class separation, (d) proportion of individuals per class, homogeneity within the latent classes and (e) meaningfulness and interpretability of the identified classes [43, 44, 48]. The PrEP use stigma classes identified were used to construct a categorial outcome for the unadjusted and adjusted analyses of membership in latent PrEP use stigma classes.

#### Regression Analyses Predicting Latent Class Membership of PrEP Use Stigma

To assess associations between risk factors and PrEP use stigma class membership, multinomial logistic regression was used for the unadjusted and adjusted analyses. Latent class membership was the outcome variable, and we aimed to identify risk factors of membership in the classes with the lowest membership, compared to the class with the highest membership.

All analyses were done in Stata version 15.1 (StataCorp) and P < 0.05 was set to be the level of significance cut-off. Risk factors were assessed individually first, and only significant risk factors were included in adjusted analyses controlling for potential confounders.

### Ethical Considerations

Ethical approval for the study was obtained from the Human Research Ethics Committee of the University of Witwatersrand in Johannesburg, South Africa, the Medical Research Coordinating Committee of the Tanzania National Institute for Medical Research, and the ethics committee of the London School of Hygiene and Tropical Medicine.

## Results

### Exploratory Factor Analysis

Eight items were assessed. In the initial analysis, the items grouped on two factors, however, two items (Item 3: ‘I feel empowered to use PrEP’ and Item 8: ‘People will think I am behaving responsibly by taking PrEP’) were dropped due to weak loadings (< 0.4) and low item specific uniqueness. When the factor analysis was re-specified, the remaining six items loaded onto one factor representing an overall PrEP use stigma scale that demonstrated very good internal reliability (α = 0.81). The one factor solution explained 88% of the total variance (Table 1). As the six items represented one underlying construct, we created a dichotomous variable capturing agreement with any of the six PrEP use stigma items versus no agreement for our outcome measure.

**Table 1:** Rotated factor loadings for 2-factor and 1-factor solutions of PrEP use stigma items

Scale items	Two factors		One factor	Uniqueness
	Original (N = 307)		Re-specified (N = 307)	
	Factor 1	Factor 2	Factor 1: PrEP use stigma	
Item 1: I feel ashamed to use PrEP	0.6094	0.6802	0.8302	0.3107
Item 2: I feel embarrassed to use PrEP	0.6379	0.7001	0.8598	0.2607
Item 3: I feel empowered to use PrEP (*reverse coded*)^a^	0.1836	0.6105	–	–
Item 4: I think I am not following the ‘rules’ (expectations) of my community if I take PrEP to prevent HIV	0.6214	0.2179	0.6541	0.5721
Item 5: I think people will give me a hard time (such as make fun of me, or talk badly about me) if I tell them I am taking PrEP	0.8998	0.0027	0.8284	0.3138
Item 6: I think people will judge me negatively if I take PrEP	0.9037	− 0.0724	0.8026	0.3558
Item 7: I think I am at greater risk for physical violence or rape if I take PrEP	0.5925	0.2185	0.6289	0.6045
Item 8: People will think I am behaving responsibly by taking PrEP (*reverse coded*)^b^	− 0.0908	0.2748	–	–
*Cronbach’s alpha*	0.71		0.81	

^a^Item dropped to do double loading on both factors

^b^Item dropped due to poor loading (< 0.4) on both factors

### Study Population

Of the 307 AGYW, 276 (89.9%) were from South Africa while 31 (10.1%) were from Tanzania. More than half (54.2%) of study participants were aged above 20 years and the median age was 21 years (interquartile range: 19–22 years). Overall, 34% of participants agreed with any of the six PrEP use stigma items derived from the factor analysis (Fig. 1b). There were significant differences in reported PrEP use stigma by site, with South African participants reporting more stigma (37% compared to 9.7%) than Tanzanian participants. Participants who told their partner about their planned PrEP use reported less PrEP use stigma (24% compared to 44%) than those who did not. Higher PrEP use stigma was reported among participants who reported any fear or shame about people living with HIV (43% compared to 30%) than those who did not report such stigma (Table 2). Similar characteristics were statistically different between the PrEP stigma class membership (Table 3).

**Table 2:** Baseline characteristics and risk factor analysis of PrEP stigma among adolescent girls and young women in South Africa and Tanzania who participated in the EMPOWER trial

Characteristics	Any PrEP use stigma (N = 307)			Unadjusted logistic model	Adjusted multivariable logistic model^a^ (N = 305)
	Yes N (row %)	No N (row %)	X^2^ (df, p value)	Unadjusted odds ratio (95% CI)	Adjusted odds ratio (95% CI)
VOICE risk score^b^					
< 5	5 (41.7)	7 (58.3)		Ref	
≥ 5	98 (33.2)	197 (66.8)	3.2 (1, 0.07)	0.36 (0.11; 1.15)	
Depression—CES-D score^c^					
< 10	102 (71.3)	41 (28.7)		Ref	
≥ 10	100 (61.0)	64 (39.0)	3.6 (1, 0.06)	1.59 (0.99; 2.57)	
Ever experienced GBV					
No	65 (31.3)	143 (58.7)		Ref	
Yes	40 (40.4)	59 (59.6)	2.5 (1, 0.11)	1.49 (0.91; 2.45)	
Perceived risk of HIV					
Not worried	22 (28.2)	56 (71.8)		Ref	
Some worry	29 (32.6)	60 (67.4)	2.1 (2, 0.35)	1.23 (0.63; 2.38)	
A lot of worry	52 (37.7)	86 (62.3)		1.53 (0.84; 2.80)	
Believes PrEP prevents HIV					
No	17 (41.5)	24 (58.5)		Ref	
Yes	86 (32.6)	178 (67.4)	1.3 (1, 0.26)	0.68 (0.34; 1.34)	
Told partner about plans to use PrEP					
No	67 (43.5)	87 (56.5)		Ref	Ref
Yes	38 (24.8)	115 (75.2)	11.9 (1, 0.001)	0.43 (0.26; 0.70)**	0.41 (0.24; 0.70)**
Number of sexual partners					
1	78 (32.1)	165 (67.9)		Ref	
2+	26 (41.9)	36 (58.1)	2.2 (1, 0.15)	1.52 (0.86; 2.71)	
Transactional sex past 4 weeks					
No	23 (39.7)	35 (60.3)		Ref	
Yes	82 (32.9)	167 (67.1)	0.9 (1, 0.33)	0.75 (0.41; 1.35)	
Partner age difference^d^					
< 5 years	113 (65.7)	77 (65.3)		Ref	
≥ 5 years	41 (34.7)	77 (65.3)	0.006 (1, 0.94)	1.02 (0.62; 1.67)	
Any sexually transmitted infection					
No	70 (35.9)	125 (64.1)		Ref	
Yes	35 (31.2)	77 (60.8)	0.7 (1, 0.41)	0.01 (0.49; 1.33)	
Hazardous and harmful drinking					
No	86 (33.1)	174 (66.9)		Ref	
Yes	19 (40.4)	28 (59.6)	1.0 (1, 0.33)	1.37 (0.73; 2.60)	
Any fear and shame about PLHIV					
No	64 (29.9)	150 (70.1)	4.79 (0.03)	Ref	Ref
Yes	39 (42.9)	52 (57.1)		1.76 (1.06; 2.92)*	1.96 (1.15; 3.34)*
Any perceived HIV stigma^e,f^					
No	1 (6.7)	14 (93.3)	5.18 (1, 0.02)		
Yes	102 (35.2)	188 (64.8)			
Site^e^					
South Africa	102 (37.0)	174 (68.0)			
Tanzania	3 (9.7)	28 (90.3)	9.2 (1, 0.002)		
Age range (years)					
16–20	49 (35.5)	89 (64.5)	0.34 (1, 0.56)		
21–24	54 (32.3)	113 (67.7)			
Married/cohabiting					
No	98 (33.9)	191 (66.1)			
Yes	7 (38.9)	11 (61.1)	0.19 (1, 0.67)		
Living with					
Alone/children	28 (37.0)	46 (62.2)			
Parents/relatives	70 (34.3)	134 (65.7)	1.74 (2, 0.42)		
Partner	7 (24.1)	22 (75.9)			
Currently in school					
No	33 (28.0)	85 (72.0)			
Yes	70 (37.4)	117 (62.6)	2.9 (1, 0.89)		
Number of children					
0	68 (35.2)	125 (64.8)	0.25 (1, 0.62)		
1+	37 (32.5)	77 (67.5)			

*GBV* gender-based violence; *PrEP* pre-exposure prophylaxis; *PLHIV* people living with HIV

*P < 0.05; **P < 0.001 for logistic model

^a^In the multivariable models, individual risk factors are adjusted for the following potential confounders: study site, age range, marital status, living status, currently in school and number of children

^b^The VOICE risk score is Derived from Balkus JE, Brown E, Palanee T, Nair G, Gafoor Z, Zhang J, et al. An empiric HIV risk scoring tool to predict HIV-1 acquisition in African women. Journal of Acquired Immune Deficiency Syndromes (1999) 2016; 72(3):333–343

^c^Derived from Radloff LS. The CES-D scale: a self-report depression scale for research in the general population. Applied Psychological Measurement 1977; 1(3):385–401

^d^Only 290 respondents answered the question about partner age difference

^e^Fisher’s exact test was performed for variable as one cell had a frequency less than 5

^f^Adjusted analysis was not performed with perceived stigma as one cell had less than 5 responses

**Table 3:** Baseline characteristics of PrEP use stigma latent classes among AGYW in South Africa and Tanzania who participated in the EMPOWER trial

Characteristics	Latent Class 1 High PrEP use stigma (N = 65)	Latent Class 2 Medium PrEP use stigma (N = 98)	Latent Class 3 Low PrEP use stigma (N = 144)	X^2^ (df, p value)
	N (%)	N (%)	N (%)	
Site^a^				
South Africa	63 (96.9)	84 (85.7)	129 (89.6)	5.4 (2, 0.06)
Tanzania	2 (3.1)	14 (14.3)	15 (10.4)	
Age range (years)				
16–20	29 (44.6)	47 (48.0)	62 (43.7)	0.4 (2, 0.80)
21–24	36 (55.4)	51 (52.0)	80 (56.3)	
Married/cohabiting^a^				
No	64 (98.5)	87 (88.9)	138 (95.8)	8.1 (2, 0.02)
Yes	1 (1.5)	11 (11.2)	6 (4.2)	
Living with^a^				
Alone/children	19 (29.2)	24 (24.5)	31 (21.5)	
Parents/relatives	43 (66.2)	60 (61.2)	101 (70.2)	6.0 (4, 0.2)
Partner	3 (4.6)	14 (14.3)	12 (8.3)	
Currently in school				
No	17 (26.2)	43 (43.9)	58 (40.9)	5.7 (2, 0.06)
Yes	48 (73.8)	55 (56.1)	84 (59.1)	
Number of children				
0	48 (73.9)	55 (56.1)	90 (62.5)	5.3 (2, 0.07)
1+	17 (26.1)	43 (43.9)	54 (37.5)	
VOICE risk score^a,b^				
< 5	1 (1.5)	7 (7.1)	4 (2.8)	4.2 (2, 0.19)
≥ 5	64 (98.5)	91 (92.9)	140 (97.2)	
Depressive symptoms (CES-D score)^c^				
< 10	22 (33.8)	53 (54.1)	68 (47.2)	6.5 (2, 0.04)
≥ 10	43 (66.2)	45 (45.9)	76 (52.8)	
Ever experienced GBV				
No	44 (67.7)	69 (70.4)	95 (66.0)	0.5 (2, 0.77)
Yes	21 (32.3)	29 (29.6)	49 (34.0)	
Perceived risk of HIV				
Not worried	14 (21.6)	27 (27.6)	37 (26.6)	
Some worry	24 (36.9)	27 (27.5)	38 (26.8)	2.6 (4, 0.62)
A lot of worry	27 (41.5)	44 (44.9)	67 (47.2)	
Believes PrEP prevents HIV				
No	16 (24.6)	13 (13.3)	12 (8.5)	10.0 (2, 0.007)
Yes	49 (75.4)	85 (86.7)	130 (91.6)	
Told partner about plans to use PrEP				
No	41 (63.1)	51 (52.0)	62 (43.1)	7.4 (2, 0.03)
Yes	24 (36.9)	47 (48.0)	82 (56.9)	
Number of partners				
1	51 (79.7)	78 (79.6)	114 (79.7)	0.0006 (2, 1.00)
2+	13 (20.30	20 (20.4)	29 (20.3)	
Transactional sex in past 4 weeks				
No	12 (18.50)	15 (15.3)	31 (21.5)	1.5 (2, 0.48)
Yes	53 (81.5)	83 (84.70)	113 (78.5)	
Partner age difference^d^				
< 5 years	42 (68.9)	57 (60.0)	73 (54.5)	3.6 (2, 0.16)
≥ 5 years	19 (31.1)	38 (40.0)	61 (45.5)	
Any sexually transmitted infections				
No	44 (67.7)	65 (66.3)	86 (59.7)	1.7 (2, 0.42)
Yes	21 (32.3)	33 (33.7)	58 (40.3)	
Hazardous and harmful drinking				
No	54 (83.1)	86 (87.8)	120 (83.3)	1.0 (2, 0.59)
Yes	11 (16.9)	12 (12.2)	24 (16.7)	
Any fear and shame about PLHIV				
No	44 (67.69)	68 (69.39)	102 (71.83)	0.4 (0.82)
Yes	21 (32.31)	30 (30.61)	40 (28.17)	
Any perceived HIV stigma^a^				
No	1 (1.54)	6 (6.12)	8 (5.63)	2.0 (2, 0.38)
Yes	64 (98.46)	92 (93.88)	1.34 (94.37)	

*GBV* gender-based violence. *PrEP* pre-exposure prophylaxis; *PLHIV* people living with HIV

^a^Fisher’s exact tests were performed for variables with a frequency of less than 5 in any cell

^b^The VOICE risk score is Derived from Balkus JE, Brown E, Palanee T, Nair G, Gafoor Z, Zhang J, et al. An empiric HIV risk scoring tool to predict HIV-1 acquisition in African women. Journal of Acquired Immune Deficiency Syndromes (1999) 2016; 72(3):333–343

^c^Derived from Radloff LS. The CES-D Scale: a self-report depression scale for research in the general population. Applied Psychological Measurement 1977; 1(3):385–401

^d^Only 290 respondents answered the question about partner age difference

### Risk Factors for PrEP Use Stigma

Only two characteristics predicted any PrEP use stigma. Participants who had told their partners about their planned PrEP use were significantly less likely to report any PrEP use stigma (adjusted odds ratio [AOR] 0.45; 95% confidence interval [CI 0.26–0.78]). Conversely, participants who reported any fear or shame about people living with HIV were 96% more likely to report any PrEP use stigma (AOR: OR 1.96; 95% CI 1.15–3.34) (Table 2).

### PrEP Use Stigma Latent Classes

Applying the criterion outlined in the “Methods” section, we identified three latent classes. The three latent class solution captured homogeneous groups representing different levels of PrEP use stigma. As such, to ease interpretation we named the latent classes ‘high PrEP use stigma’ (21%; 65/307), which had 78% of its class membership agreeing with any of the six PrEP use stigma items, ‘medium PrEP use stigma’ (32%; 98/307), with 27% of class membership reporting PrEP use stigma and ‘low PrEP stigma’ (47%; 144/307), with 19% of class membership reporting any PrEP use stigma (Table 3).

Excluding characteristics with less than five responses in a cell, we observed significant differences across the latent classes for three variables in bivariate analyses: depressive symptoms, belief that PrEP prevents HIV, and having told a partner about plans to use PrEP (Table 3).

### Factors Associated with Latent Classes of PrEP Stigma

Results from the adjusted multinomial logistic regression indicated that AGYW who told their partner about taking PrEP were more likely to report low PrEP use stigma than those who did not (adjusted relative risk ratios (aRRR) 0.44, 95% [CI] 0.23–0.82). Likewise, AGYW who believed PrEP prevents HIV were more likely to report low PrEP use stigma than those who did not (aRRR 0.31, 95% CI 0.14–0.72) (Table 4).

**Table 4:** Risk factor analysis of high and medium PrEP use stigma latent classes, compared to the low PrEP use stigma latent class, among AGYW in South Africa and Tanzania who participated in the EMPOWER trial

Characteristics	Latent Class 1 High PrEP use stigma		Latent Class 2 Medium PrEP use stigma
	Unadjusted model RRR^a^ (95% CI) (N = 305)	Adjusted model^a^ RRR (95% CI) (N = 305)	Unadjusted model^c^ RRR (95% CI) (N = 305)
CES-D score^b^			
< 10	Ref		Ref
≥ 10	1.75 (0.95; 3.22)		0.76 (0.45; 1.27)
Ever experienced GBV			
No	Ref		Ref
Yes	0.93 (0.50; 1.73)		0.81 (0.47; 1.42)
Perceived risk of HIV			
Not worried	Ref		Ref
Some worry	1.67 (0.75; 3.71)		1.07 (0.50; 2.28)
A lot of worry	0.97 (0.48; 1.96)		0.90 (0.48; 1.68)
Believes PrEP prevents HIV			
No	Ref	Ref	Ref
Yes	0.31 (0.12; 0.64)*	0.31 (0.14; 0.72)*	0.60 (0.26; 1.39)
Told partner about plans to use PrEP			
No	Ref	Ref	Ref
Yes	0.44 (0.24; 0.81)*	0.44 (0.23; 0.82)*	0.70 (0.42; 1.17)
Number of partners			
1	Ref		Ref
2+	1.00 (0.48; 2.09)		1.01 (0.53; 1.91)
Transactional sex last 4 weeks			
No	Ref		Ref
Yes	1.21 (0.58; 2.54)		1.52 (0.77; 2.99)
Partner age difference			
< 5 years	Ref		Ref
≥ 5 years	0.5 (0.29; 1.03)		0.80 (0.47; 1.36)
Any sexually transmitted infections			
No	Ref		Ref
Yes	0.71 (0.39; 1.31)		0.75 (0.38; 1.31)
Hazardous and harmful drinking			
No	Ref		Ref
Yes	1.02 (0.47; 2.23)		0.70 (0.33; 1.47)
Any fear and shame about people living with HIV			
No	Ref		Ref
Yes	1.22 (0.64; 2.30)		1.13 (0.64; 1.98)

*RRR* relative risk ratio; *GBV* gender-based violence; *PrEP* pre-exposure prophylaxis

*P < 0.01

^a^In the multivariable models, individual risk factors are adjusted for the following potential confounders: study site, age range, marital status, living status, currently in school and number of children

^b^Derived from Radloff LS. The CES-D Scale: a self-report depression scale for research in the general population. Applied Psychological Measurement 1977; 1(3):385–401

^c^Mutivariable logistic regression results are not shown for latent class 2, as none of the risk factors were significant in the simple multinomial logistic regressions

## Discussion

We explored dimensions of PrEP use stigma among AGYW in SSA using two approaches. Factor analysis yielded one dimension consisting of six items capturing both fear and shame about PrEP use and anticipated stigma and discrimination linked with PrEP use. The resulting brief, PrEP use stigma scale (B-PSS) had very good internal reliability and is recommended for use in future studies with similar populations. Latent class analysis yielded three classes, reflecting high, medium and low reported PrEP use stigma, respectively. Adjusted, multivariable analysis with the 6-item scale and the latent classes identified similar correlates. Namely, disclosure of PrEP use to sexual partners and belief that PrEP prevents HIV appear to have a protective effect. Conversely, fear and shame about people living with HIV may increase PrEP use stigma considerably.

Ours is the first scale to be tested among AGYW in SSA. Like the 11-item scale developed by Klein and Washington [28], our scale was unidimensional, and focused mainly on capturing anticipated stigma related to PrEP use. While we also included items to capture personal attitudes towards PrEP use, only the negatively framed items were ultimately included due to poor factor loadings. This phenomenon has been observed previously in the HIV stigma measurement field, where positively framed items often have little variance (e.g., agreement is very high), so they are dropped from multi-item scales [49]. However, including more positively framed items could have yielded a distinct dimension, as was observed in the scale developed by Mustanski et al. [27].

While other domains of PrEP-related stigma, such as PrEP stigma stereotypes and anticipated disapproval of PrEP use, have emerged in the literature as significant barriers to PrEP initiation [5, 28], our scale was developed for and tested among AGYW who were participating in a PrEP demonstration trial in which they were offered, and the majority initiated, PrEP. As such, we assessed anticipated PrEP use stigma and personal attitudes about PrEP use. It is likely that different dimensions of PrEP-related stigma will be more relevant based on the context and study population. For example, qualitive findings from our study [25] and similar studies in SSA [7] now suggest that PrEP-HIV stigma and PrEP-sexual stigma are key concerns among AGYW initiating PrEP in SSA. Thus, future research is needed to develop and validate measures capturing these domains, in addition to PrEP use stigma, among AGYW in SSA.

We found that disclosure of planned PrEP use to a sexual partner at baseline was significantly associated with less anticipated PrEP use stigma as measured by our scale. Given the nascent stage of research on PrEP use stigma, we did not find similar research to compare our findings with. However, Phillips et al. reported that young men who have sex with men and transgender women in the US who disclosed PrEP use to relatives received more support from family and friends to continue on PrEP [50]. Likewise, qualitative data from the EMPOWER study suggested that our participants disclosed PrEP use to secure support from their family and/or to advocate and encourage their at-risk peers to take PrEP [25, 51]. This suggests that social support plays an important role in mitigating stigma, and in particular partner support. Similar to previous research [52], those with any fear and shame about people living with HIV at baseline were significantly more likely to report anticipated PrEP use stigma.

We were also interested to explore whether the combinations of stigmatizing items respondents agreed with mattered more so than their reported PrEP use stigma, in terms of risk factors for PrEP use stigma among our participants. Indeed, we found that one additional protective factor emerged in the adjusted multinomial analysis of the three latent classes, or patterns of responses that emerged from the LCA. In addition to disclosure of planned PrEP use to a partner, belief that PrEP use prevented HIV was also protective against anticipated PrEP use stigma. Our finding is similar to a study conducted in China among MSM, which found that higher perceived benefits of PrEP increased the likelihood of PrEP uptake, suggesting the importance of PrEP sensitization prior to rolling out PrEP programs [53].

A few limitations to our study should be noted. Firstly, these analyses were cross-sectional, as we only had data on PrEP use stigma at one time point (i.e. endline), therefore, we could not establish causality of the observed differences in PrEP use stigma by participant characteristics or study arm. As we only assessed PrEP use stigma at endline, after most participants had initiated PrEP, it is possible that the level of PrEP use stigma may be higher at the start of PrEP use. In addition, our findings may not be relevant for PrEP-naïve AGYW at high risk for HIV in SSA who are not yet aware of PrEP. Future research should assess PrEP use stigma at multiple time points to assess longitudinal changes over time. Secondly, our measures assessed personal attitudes about PrEP use and anticipation of PrEP use stigma, as opposed to actual experiences of PrEP use stigma. Future longitudinal studies are needed to examine the frequency and correlates of experienced PrEP use stigma as AGYW initiate and continue using PrEP. Thirdly, our sample size was limited given the nature of our PrEP demonstration trial, which was designed to pilot and assess an empowerment intervention on PrEP uptake and continuation among AGYW. Enrolment in Mwanza, Tanzania was considerably lower than in Johannesburg, South Africa. Therefore, our findings should be interpreted with caution, as we may not have had sufficient power to identify risk factors of PrEP use stigma. Lastly, our study sample was specific to urban AGYW in Johannesburg and AGYW who worked in bars in Mwanza, thus our findings may not be generalizable to all AGYW in SSA. Despite these limitations, our study is the first to report risk factors of PrEP use stigma among AGYW in SSA and will inform future research with this population.

## Conclusion

Given widespread PrEP roll-out for AGYW in SSA and the potential barrier PrEP use stigma poses to uptake and continuation, a valid tool to assess the prevalence of PrEP use stigma and understand key risk and protective factors is needed. Our 6-item scale, the brief PrEP use Stigma Scale (B-PSS), capturing both personal attitudes about PrEP use and anticipated PrEP use stigma and discrimination, is recommended for use in future studies with similar populations. While the majority of AGYW in our study did not anticipate any PrEP use stigma, one in three did. This suggests the need for routine screening for PrEP use stigma among AGYW who could benefit from PrEP, and the development of targeted interventions to mitigate PrEP use stigma, particularly among AGYW who report fear and shame of people living with HIV, are hesitant to disclose their PrEP use to partners, and who do not believe that PrEP prevents HIV. Such interventions could include peer-mentorship, HIV prevention support groups [29] or one-on-one counseling with a health educator, ideally implemented in adolescent/youth friendly health clinics with appropriate safe spaces for learning and engagement.

## Data Availability

Study data are available upon request.

## Code Availability

STATA code for the analysis is available upon request.
